# Retraction: Endometriosis and pregnancy: The illusion of recovery

**DOI:** 10.1371/journal.pone.0338986

**Published:** 2025-12-18

**Authors:** The PLOS One Editors

The *PLOS One* Editors retract this article [1] because it was identified as one of a series of submissions for which we have concerns about compromised peer review.

The Editors have no evidence of any author involvement in the peer review concerns.

All authors did not agree with the retraction.
